# Supplementary material for: Inhibition of interleukin-1β reduces myelofibrosis and osteosclerosis in mice with JAK2-V617F driven myeloproliferative neoplasm
Source: Nat Commun. 2022 Sep 13;13:5346. doi: 10.1038/s41467-022-32927-4 (PMC9470591; doi:10.1038/s41467-022-32927-4)
Supplement: Supplementary file 1 — Supplementary Information [file 41467_2022_32927_MOESM1_ESM.pdf]

Supplementary Figure 1 (related to Figure 1)

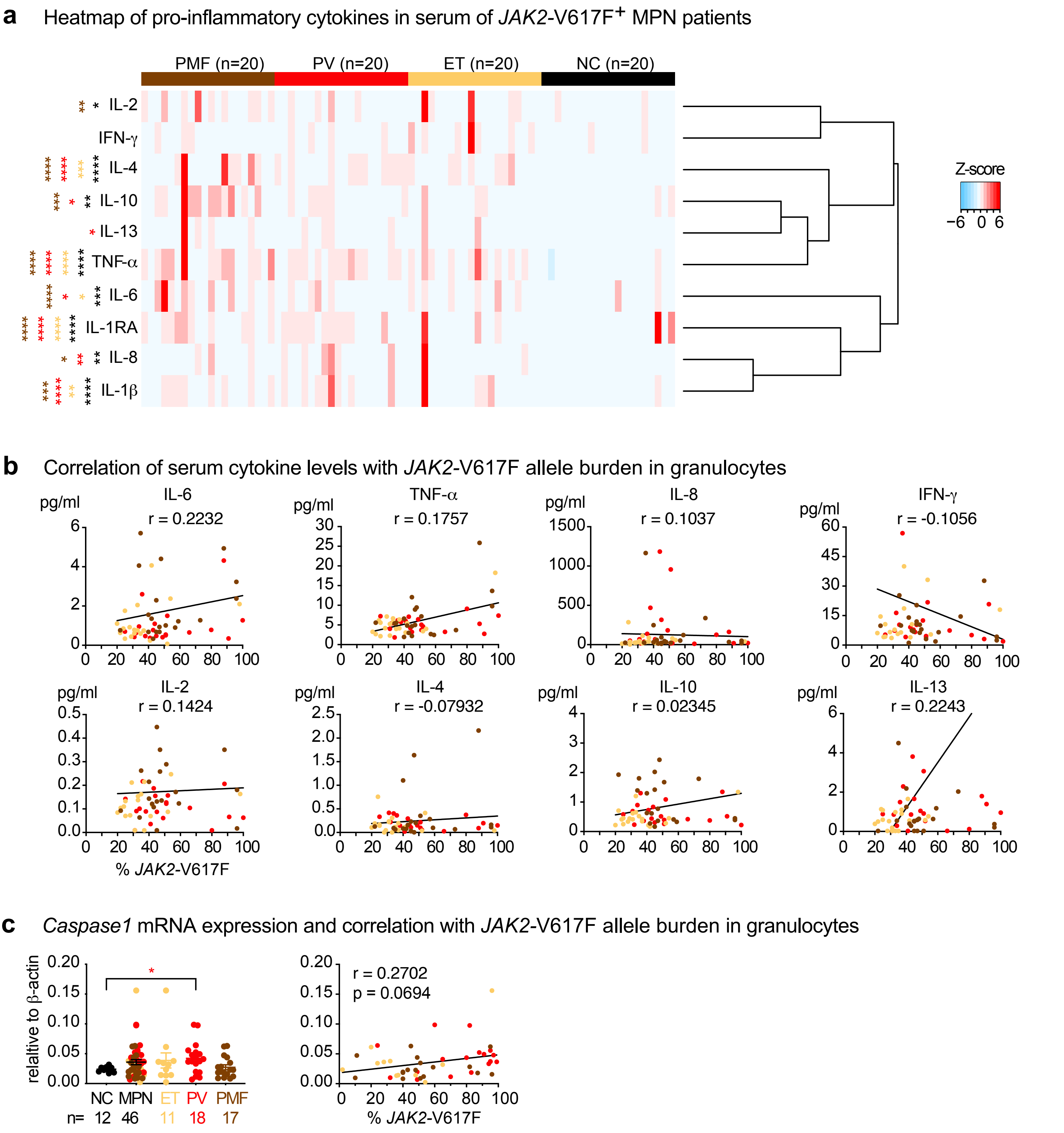

Supplementary Figure 1. Proinflammatory serum cytokines in *JAK2*-V617F-positive MPN patients. a, Heatmap showing the inflammatory cytokine levels in the serum of nomal controls (NC, n=20) and MPN patients (n=60); essential thrombocythemia (ET, n=20), polycythemia vera (PV, n=20), primary myelofibrosis (PMF, n=20). The color bars indicate different disease groups. Heatmap shows Z scores. Two-tailed non-parametric unpaired Mann-Whitney t-test was performed for p values. Black asterisk for comparison of NC vs MPN; yellow for NC vs ET; red for NC vs PV and brown for NC vs PMF. **b,** Graphs showing correlation of pro-inflammatory cytokine levels in serum with % *JAK2*-V617F in peripheral blood granulocytes. Spearman correlation (r) and unpaired two-tailed t- test was performed. **c,** *Caspase1* mRNA expression relative to β-actin in peripheral blood granulocytes of NC (n=12) and MPN patients (n=46); ET (n=11), PV (n=18), PMF (n=17). Two-tailed non-parametric unpaired Mann-Whitney t-test was performed for p values. Correlation between Caspase1 mRNA expression and % *JAK2*-V617F in peripheral blood granulocytes. Spearman correlation (r) and two-tailed t- test was performed. All data are presented as mean ± SEM. *P < .05; **P < .01; ***P < .001; ****P < .0001. Source data and exact p values are provided as a Source Data file.

Supplementary Figure 2 (related to Figure 1)

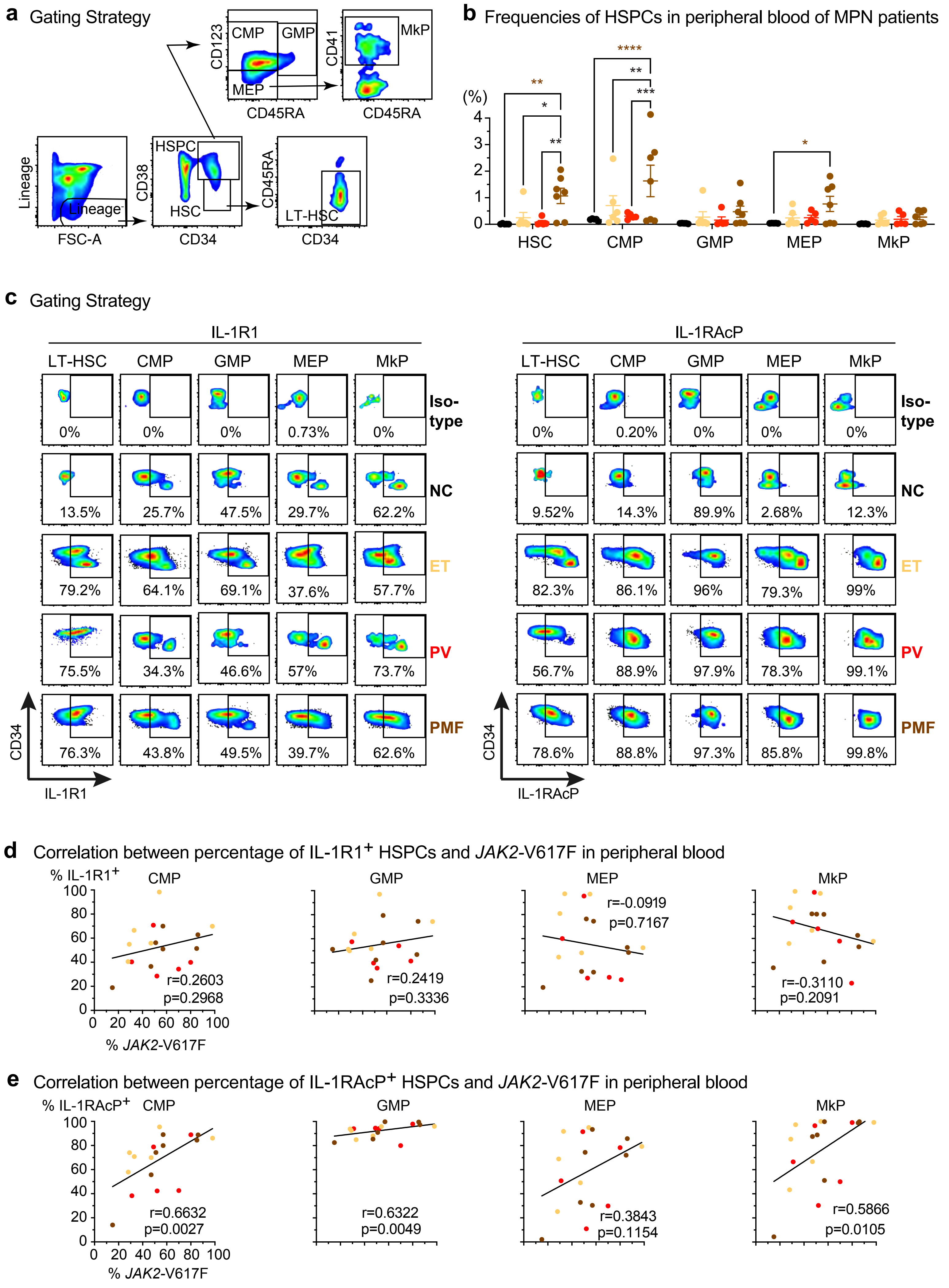

Legend to Supplementary Figure 2. Expression of IL-1 receptors on peripheral blood HSCs and HSPCs in MPN patients. a, Gating strategy for hematopoietic stem cells (HSCs) and lineage committed hematopoietic stem and progenitor cells (HSPCs) including common myeloid progenitors (CMP), granulocyte macrophage progenitor (GMP), megakaryocyte erythroid progenitor (MEP) and megakaryocyte progenitor (MkP) in peripheral blood mononuclear cells from NC and MPN patients (ET, PV and PMF). **b,** Frequencies of HSCs and HSPCs in peripheral blood of NC (n=5), ET (n=6), PV (n=5) and PMF (n=7). Two-way ANOVA was performed for statistical comparisons. **c,** Representative plots showing the gating strategy and expression patterns of interleukin 1 receptor type 1 (IL1R1), interleukin 1 receptor accessory protein (IL1RAcP) and isotype control on HSC, CMP, GMP, MEP and MkP from NC and MPN patients. **d,** Correlation (r) and significance (p) between % *JAK2*-V617F in peripheral blood granulocytes and percentages of IL-1R1+ HSPCs in the peripheral blood of NC (n=5), ET (n=6), PV (n=5) and PMF (n=7). **e,** Correlation (r) and significance (p) between % *JAK2*-V617F in peripheral blood granulocytes and percentages of IL-1RAcP+ HSPCs in the peripheral blood of NC (n=5), ET (n=6), PV (n=5) and PMF (n=7). Spearman correlation (r) and two-tailed t- test was performed for correlation analysis in **d** and **e**. All data are presented as mean ± SEM. *P < .05; **P < .01; ***P < .001; ****P < .0001. Source data and exact p values are provided as a Source Data file.

Supplementary Figure 3 (related to Figure 1)

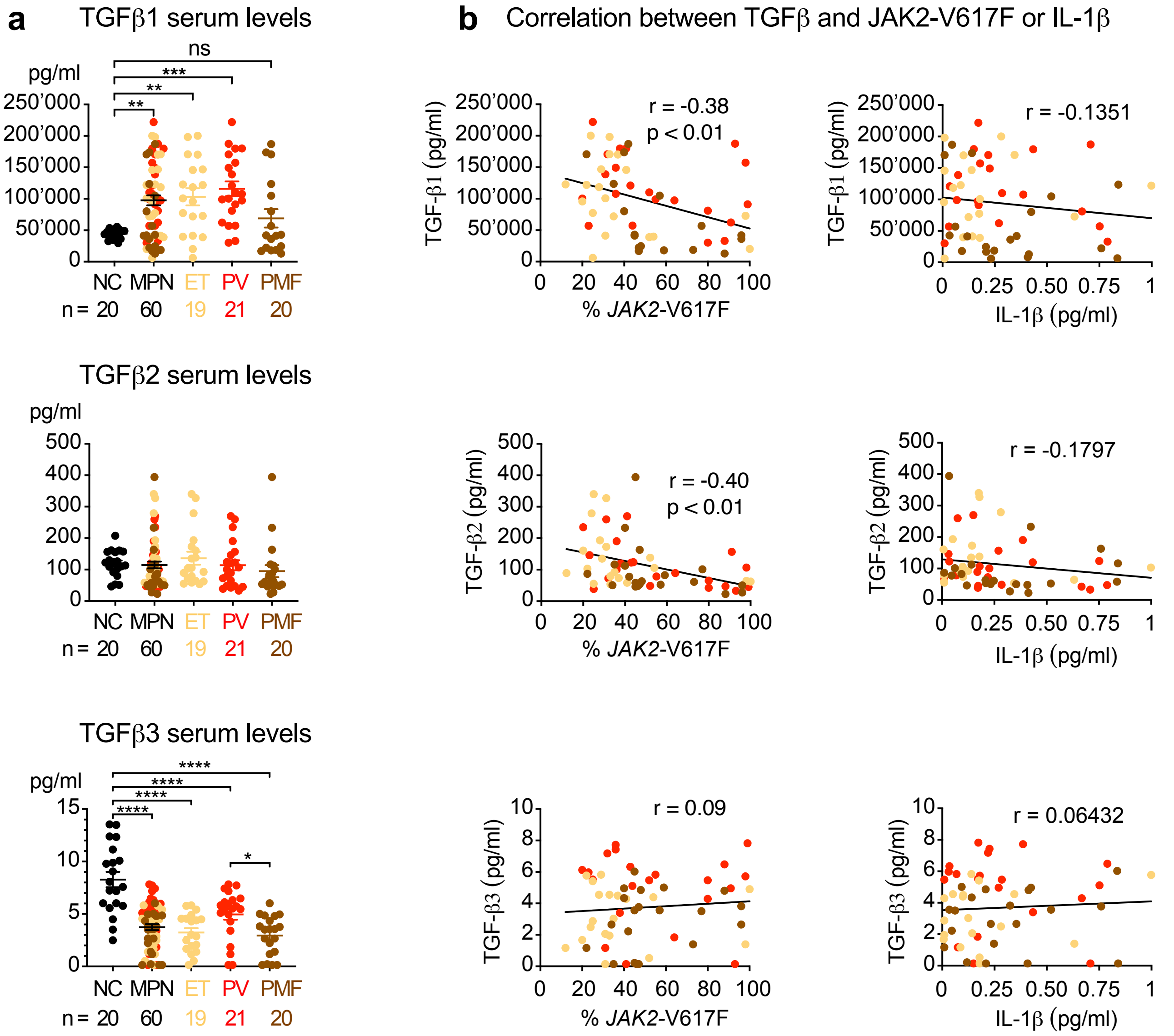

Supplementary Figure 3. TGF-β serum levels in correlation with *JAK2*-V617F allele burden and IL-1β serum levels in MPN patients. a, Graph showing serum TGF-β1/2/3 levels (pg/ml) in normal controls (NC; n=20) and MPN patients (n=60); ET (n=19), PV (n=21), PMF (n=20) (left). Ordinary one-way ANOVA with Tukey's multiple comparison tests were performed for statistical comparisons. **b,** Correlation (r) and significance (p) between % *JAK2*-V617F in peripheral blood granulocytes and serum TGF-β1/2/3 levels in MPN patients (left panel). Correlation (r) between serum TGF-β1/2/3 levels with serum IL-1-β levels in MPN patients (right panel). Peason correlation (r) and two-tailed t- test was performed for correlation analyses. All data are presented as mean ± SEM. *P < .05; **P < .01; ***P < .001; ****P < .0001. Source data and exact p values are provided as a Source Data file.

Supplementary Figure 4 (related to Figure 3)

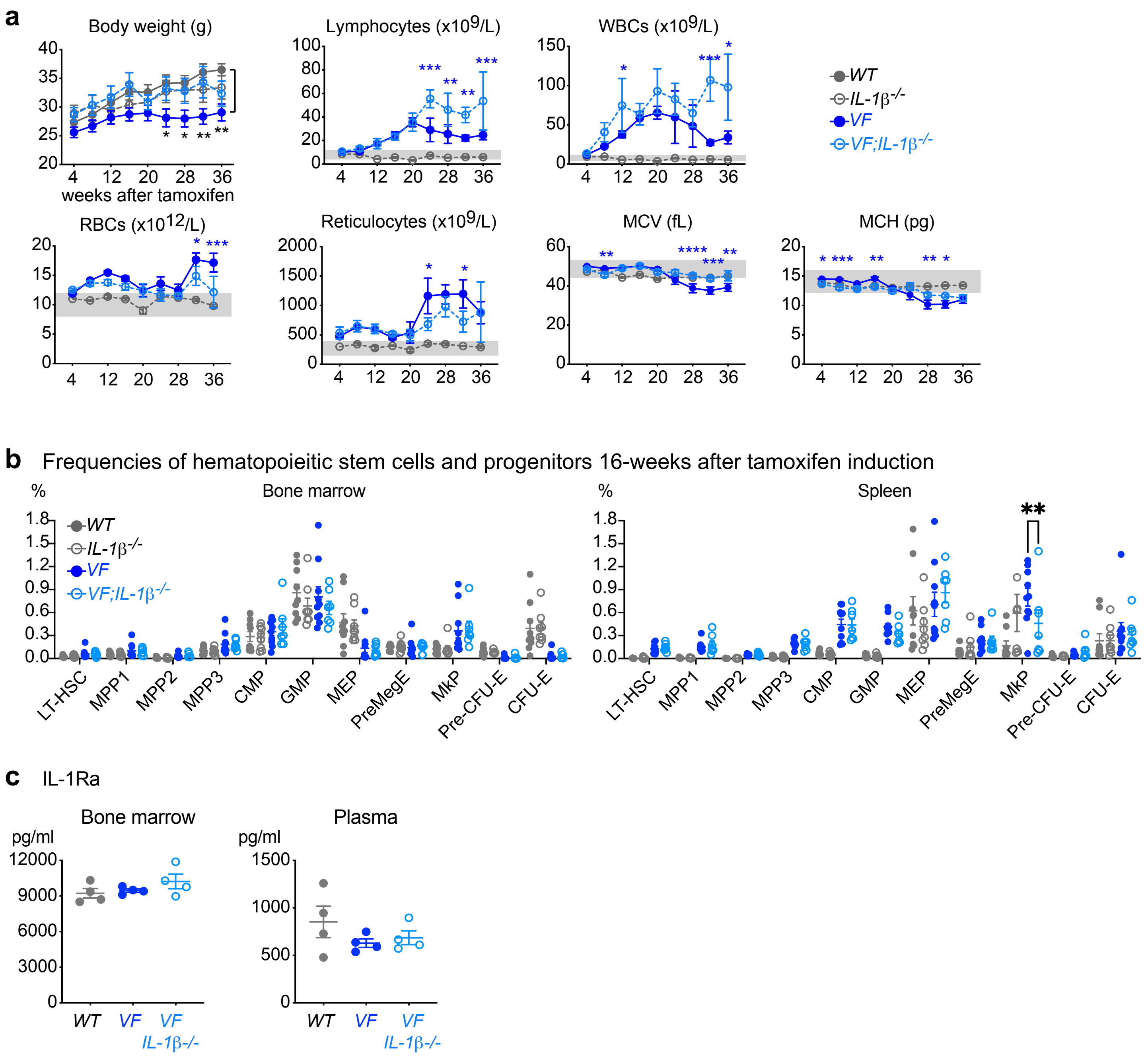

Supplementary Figure 4. Phenotype of non-transplanted *VF;IL-1β*$^{-/-}$ mice. a, Graphs showing the time course of body weights and complete blood counts after tamoxifen induction. Wildtype (*WT*; n=9), IL-1β knock-out (*IL-1β*$^{-/-}$; n=11), Scl;Cre;V617F (*VF*; n=18) and Scl;Cre;V617F; IL-1β knock-out (*VF;IL-1β*$^{-/-}$; n=13) mice were induced with tamoxifen and disease kinetics were followed for 36 weeks. Two-way ANOVA with Tukey's test was performed for multiple comparisons. **b,** Bar graphs showing the frequencies of HSCs and HSPCs in bone marrow (BM) and spleen of *WT* (n=9), *IL-1β*$^{-/-}$ (n=8), *VF* (n=10) and *VF;IL-1β*$^{-/-}$ (n=7) mice at 16 weeks after tamoxifen induction. Multiple unpaired two-tailed t-tests were performed for multiple comparisons. **c,** IL-1Ra levels in BM and plasma of *WT* (n=4), *VF* (n=4) and *VF;IL-1β*$^{-/-}$ (n=4) mice at 16 weeks after tamoxifen induction. All data are presented as mean ± SEM. *P < .05; **P < .01; ***P < .001; ****P < .0001. Source data and exact p values are provided as a Source Data file.

Supplementary Figure 5 (related to Figure 3)

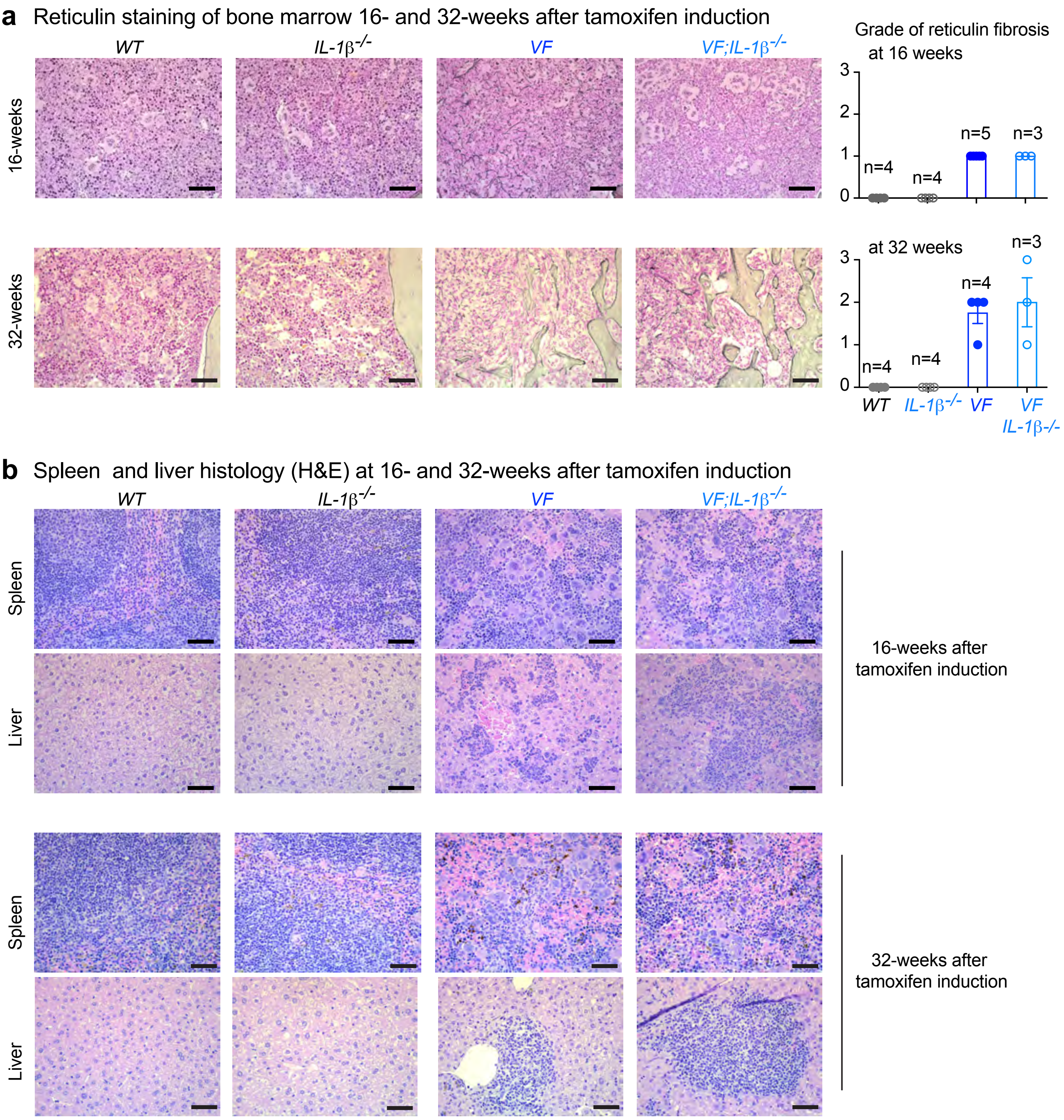

Supplementary Figure 5. Histology of non-transplanted *VF;IL-1*β$^{-/-}$ mice. a, Representative images of reticulin fibrosis in bone marrow (BM) are shown at 16-weeks (upper panel) and 32-weeks (lower panel) after tamoxifen induction. Histological grade of reticulin fibrosis in the BM is illustrated in the bar graph (right). All data are presented as mean ± SEM. *P < .05; **P < .01; ***P < .001; ****P < .0001. **b,** Representative images of spleen and liver histology (H&E staining) are shown at 16-weeks (*WT*; n=4, *IL-1β*$^{-/-}$;n=4, *VF*; n=5 and *VF;IL-1β*$^{-/-}$; n=3) and 32-weeks (*WT*; n=4, *IL-1β*$^{-/-}$;n=4, *VF*; n=4 and *VF;IL-1β*$^{-/-}$; n=3) after tamoxifen induction. Scale bar is 20 μm. Similar images were obtained with other biologically independent mice in each genotype in **a** and **b**.

Supplementary Figure 6 (related to Figure 4)

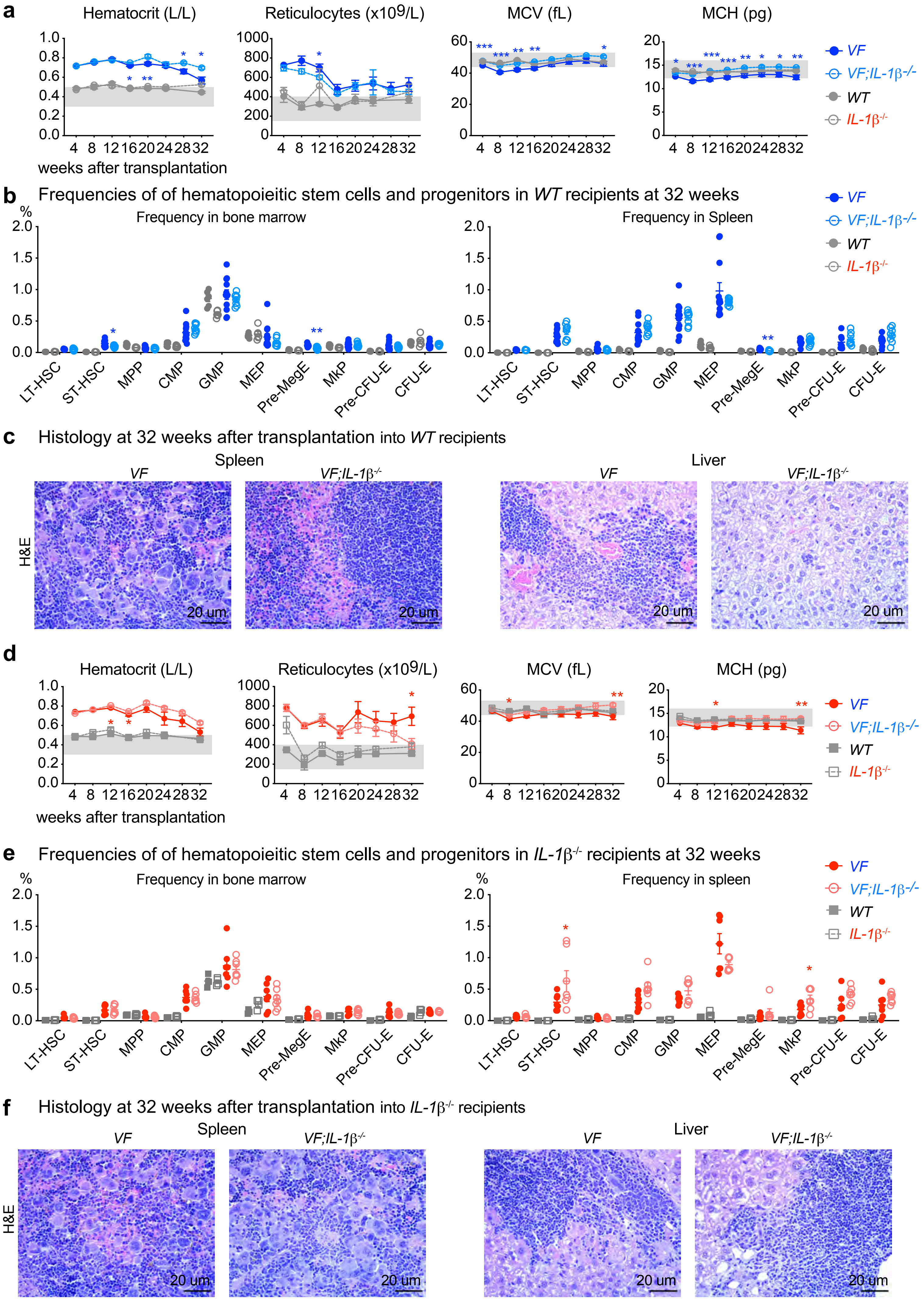

Legend to Supplementary Figure 6 (related to Figure 4)

Supplementary Figure 6. Loss of *IL-1β* in JAK2-V617F mutant cells reduces MPN symptom burden and myelofibrosis. a, Peripheral blood count of the red cell parameters after transplantation into WT recipients are shown. n=15 biologically independent mice per group. **b,** Bar graphs showing the frequencies of HSCs and HSPCs in BM and spleen of WT recipients transplanated with *WT* (n=6), *IL-1β-/-* (n=6), *VF* (n=12) or *VF;IL-1β-/-* (n=8) bone marrow. **c,** Representative images of spleen and liver histology (H&E staining) are shown at 36 weeks after transplantation in *WT* recipients transplanated with *WT* (n=6), *IL-1β-/-* (n=6), *VF* (n=12) or *VF;IL-1β-/-* (n=8) bone marrow. **d,** Peripheral blood count of the red cell parameters after transplantation into IL-1β-/- recipients are shown. n=15 biologically independent mice per group. **e,** Bar graphs showing the frequencies of HSPCs in BM and spleen of IL-1β-/- recipients transplanated with *WT* (n=4), *IL-1β-/-* (n=4), *VF* (n=7) or *VF;IL-1β-/-* (n=7) bone marrow. **f,** Representative images of spleen and liver histology (H&E staining) are shown at 36 weeks after transplantation in IL-1β-/- recipients transplanated with *WT* (n=4), *IL-1β-/-* (n=4), *VF* (n=7) or *VF;IL-1β-/-* (n=7) bone marrow. All data are presented as mean ± SEM. Statistical significances were determined by Two-tailed unpaired multiple t-tests without correction for multiple comparisons in **a**, **b**, **d** and **e**. Similar results were obtained with other mice of each genotype in **c** and **f**. *P < .05; **P < .01; ***P < .001; ****P < .0001. Source data and exact p values are provided as a Source Data file.

Supplementary Figure 7 (related to Figure 4)

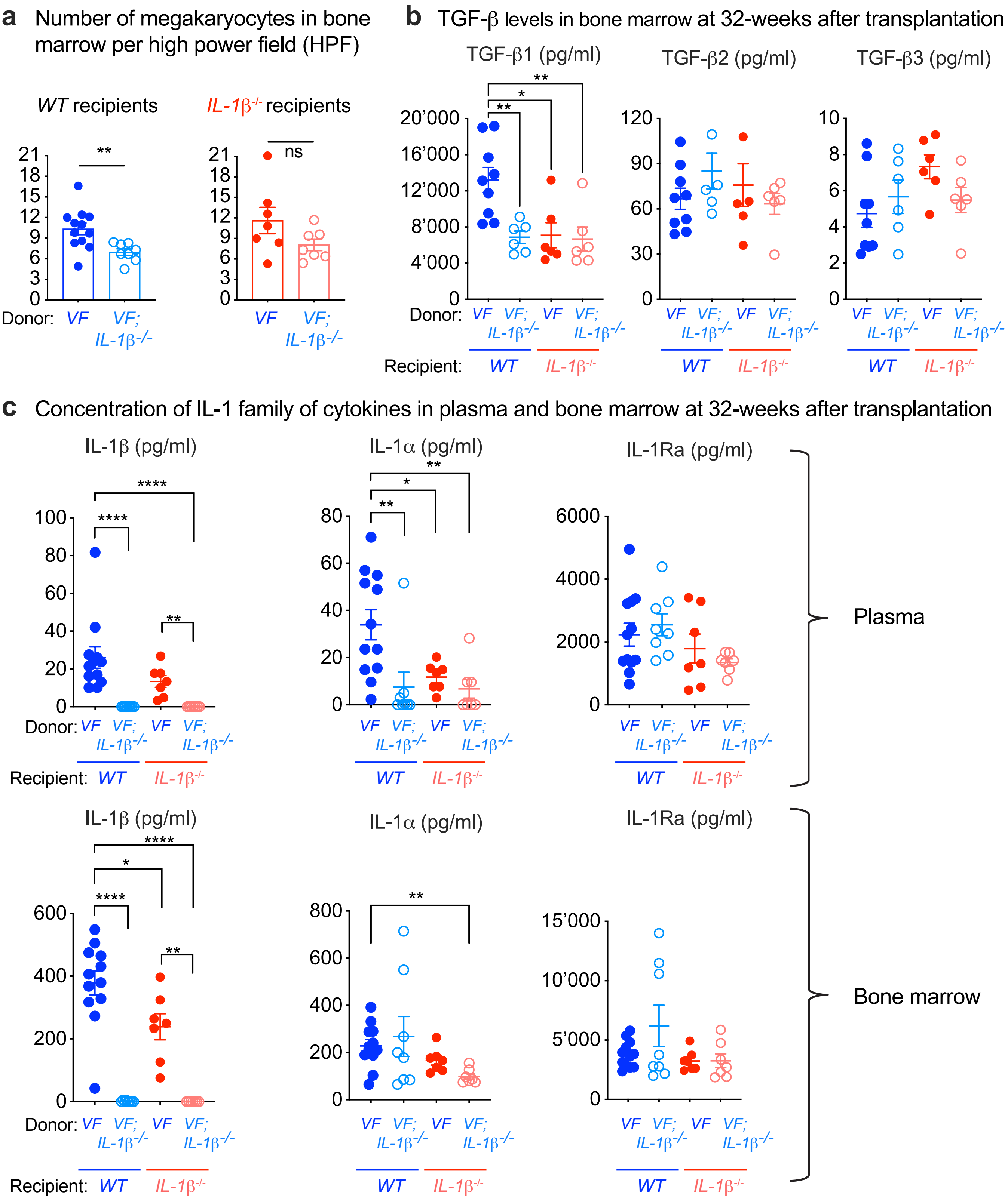

Supplementary Figure 7. Level of TGF-β and IL-1 cytokines in *WT* and *IL-1*β$^{-/-}$ recipients transplanted with *VF* or *VF;IL-1*β$^{-/-}$ bone marrow. a, Bar graphs show the number of megakaryocytes per high power visual field (HPF) at magnification 400x in *WT* recipients transplanted with *VF* (n=12) or *VF;IL-1β*$^{-/-}$ (n=8) bone marrow and *IL-1β*$^{-/-}$ recipients transplanted with *VF* (n=7) or *VF;IL-1β*$^{-/-}$ (n=7) bone marrow. Ten HPF were counted for each mouse and each dot represents the average number of megakaryocytes per 10 HPF. **b,** TGF-β cytokine levels in bone marrow of *WT* recipients transplanted with *VF* (n=9) or *VF;IL-1β*$^{-/-}$ (n=6) bone marrow and *IL-1β*$^{-/-}$ recipients transplanted with *VF* (n=5) or *VF;IL-1β*$^{-/-}$ (n=6) bone marrow (right panel). One-way ANOVA with Tukey's multiple comparison test was perfored for statistical comparisons between groups. **c,** Graphs show IL-1β, IL-1α, and IL-1Ra levels of *WT* recipients transplanted with *VF* (n=12) or *VF;IL-1β*$^{-/-}$ (n=8) bone marrow and *IL-1β*$^{-/-}$ transplanted with *VF* (n=7) or *VF;IL-1β*$^{-/-}$ (n=7) bone marrow. Statistical significance was determined by Two-tailed unpaired non-parametric Mann-Whitney t-test. All data are presented as mean ± SEM. *P < .05; **P < .01; ***P < .001; ****P < .0001. Source data and exact p value are provided as a Source Data file.

Supplementary Figure 8 (related to Figure 4)

Endosteal areas of bone marrow at 32-weeks after transplantation, Gömöri staining for reticulin fibers

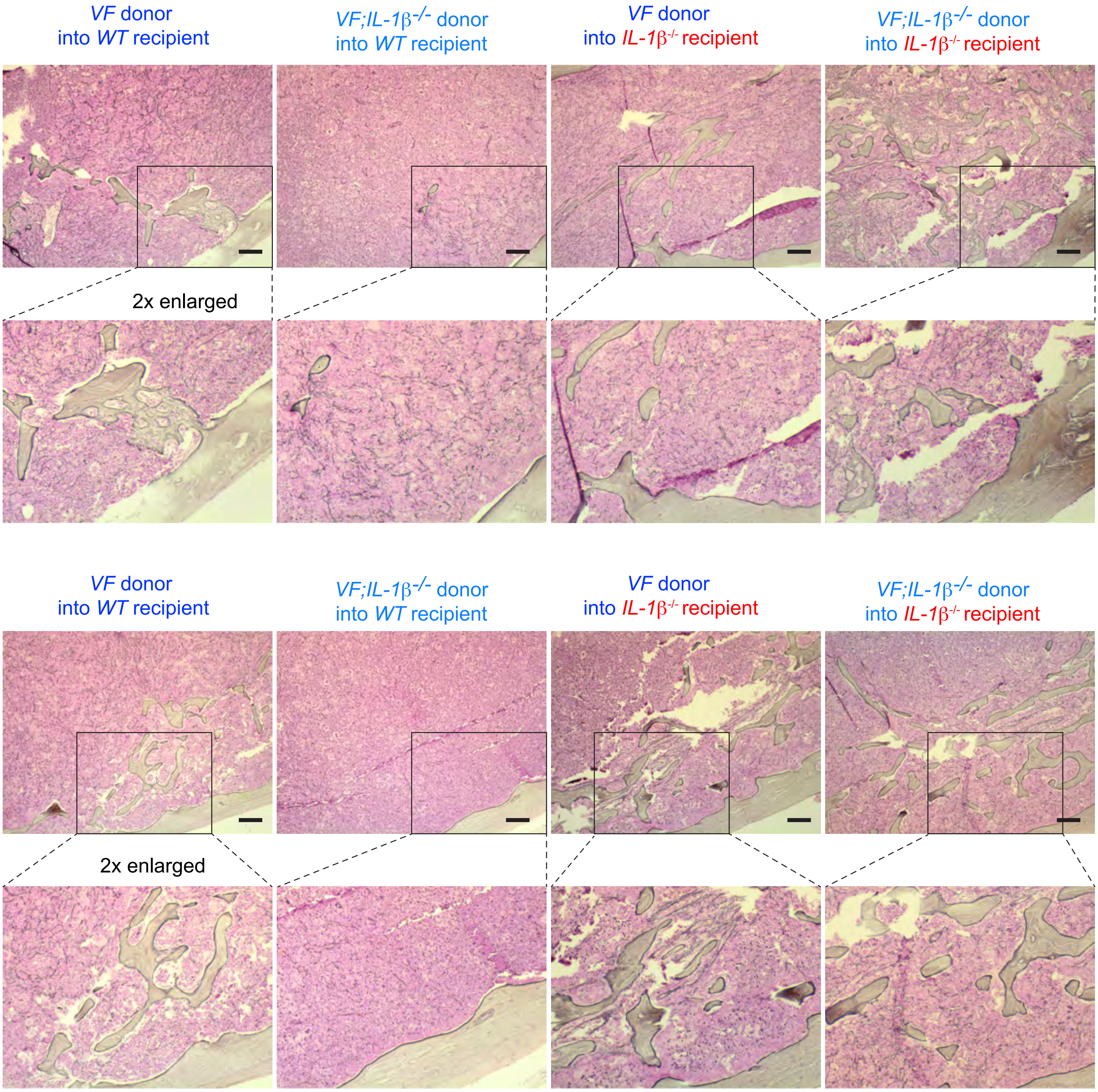

Supplementary Figure 8. Reticulin fibrosis in bone marow of *WT* and *IL-1β*$^{-/-}$ recipients transplanted with *VF* and *VF;IL-1β*$^{-/-}$ bone marrow. Representative images of reticulin fibrosis in the endosteal areas of the bone marrow from two mice per genotype are shown. Scale bar is 50 μm. Similar results were obtained in other biologically independent mice for each genotype.

Supplementary Figure 9 (related to Figure 5)

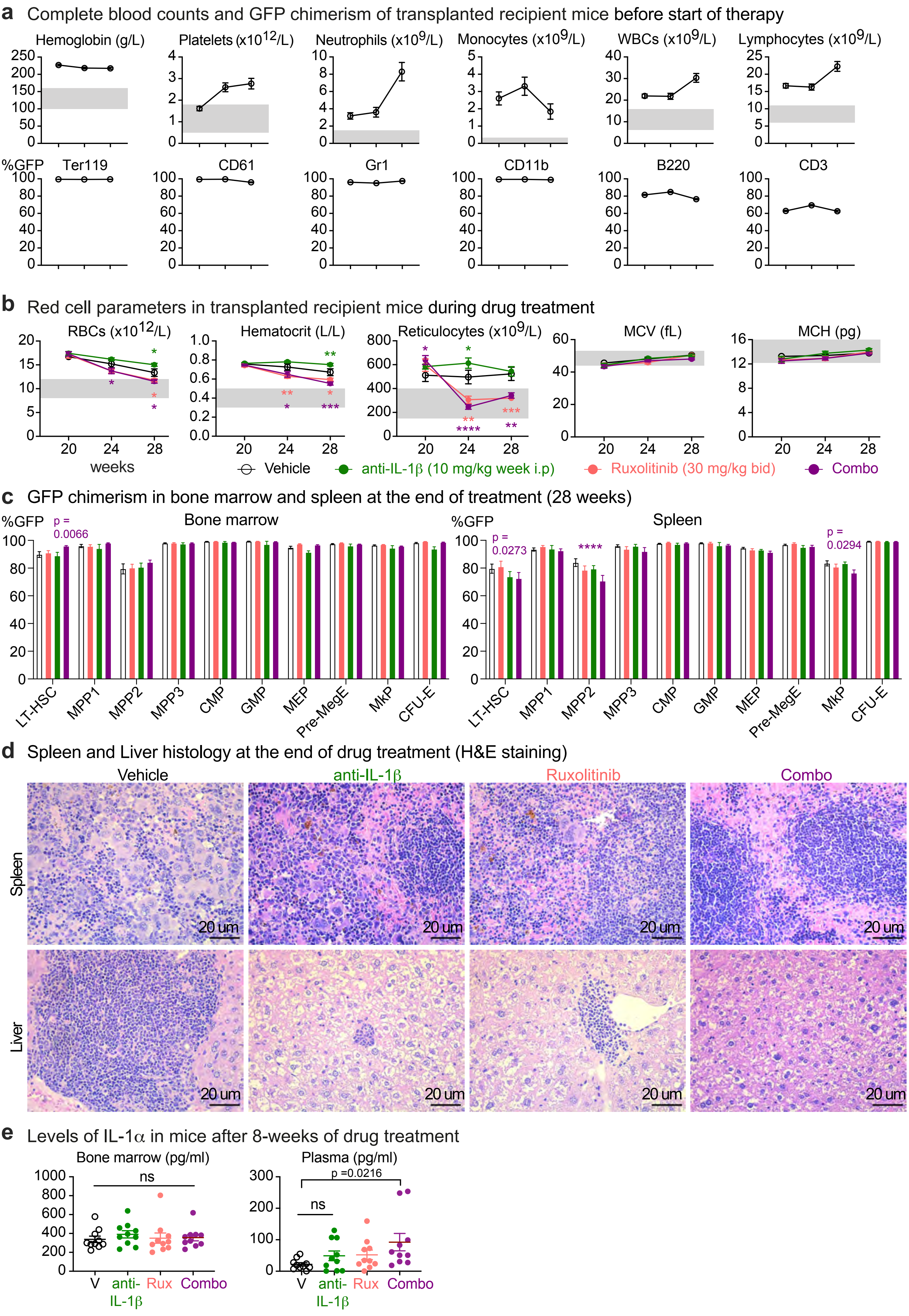

Legend to Supplementary Figure 9 (related to Figure 5)

Supplementary Figure 9. Pharmacological inhibition of IL-1β in MPN mice. a, Blood counts and GFP chimerism in peripheral blood before starting the therapy in mice (n=48) at week 12, 16 and 20 after transplantation. **b,** Red cell parameters during drug treatment. Vehicle (n=12); Rux (n=12); anti-IL-1β (n=12); combo (n=12). Two-way ANOVA followed by uncorrected Fisher's LSD test was performed for comparison with vehicle treated group. **c,** GFP chimerism in HSCs and HSPCs in the bone marrow and spleen at the end of drug treatment. Vehicle (n=12); Rux (n=11); anti-IL-1β (n=12); combo (n=12). Two-way ANOVA followed by Dunnett's multiple comparisons test was performed. **d,** Representative images of spleen and liver histology (H&E staining) after 8 weeks of drug treatment. Vehicle (n=12); Rux (n=11); anti-IL-1β (n=12); combo (n=12). Similar images were obtained with other biologically independent mice in each genotype in **d**. **e,** IL-1α levels in bone marrow and plasma of mice after 8-weeks of drug treatment. Vehicle (n=10); Rux (n=10); anti-IL-1β (n=10); combo (n=10). Two-tailed unpaired t-tests were performed for statistical comparisons. All data are presented as mean ± SEM. *P < .05; **P < .01; ***P < .001; ****P < .0001. Source data and exact p values are provided as a Source Data file.

Supplementary Figure 10 (related to Figure 5)

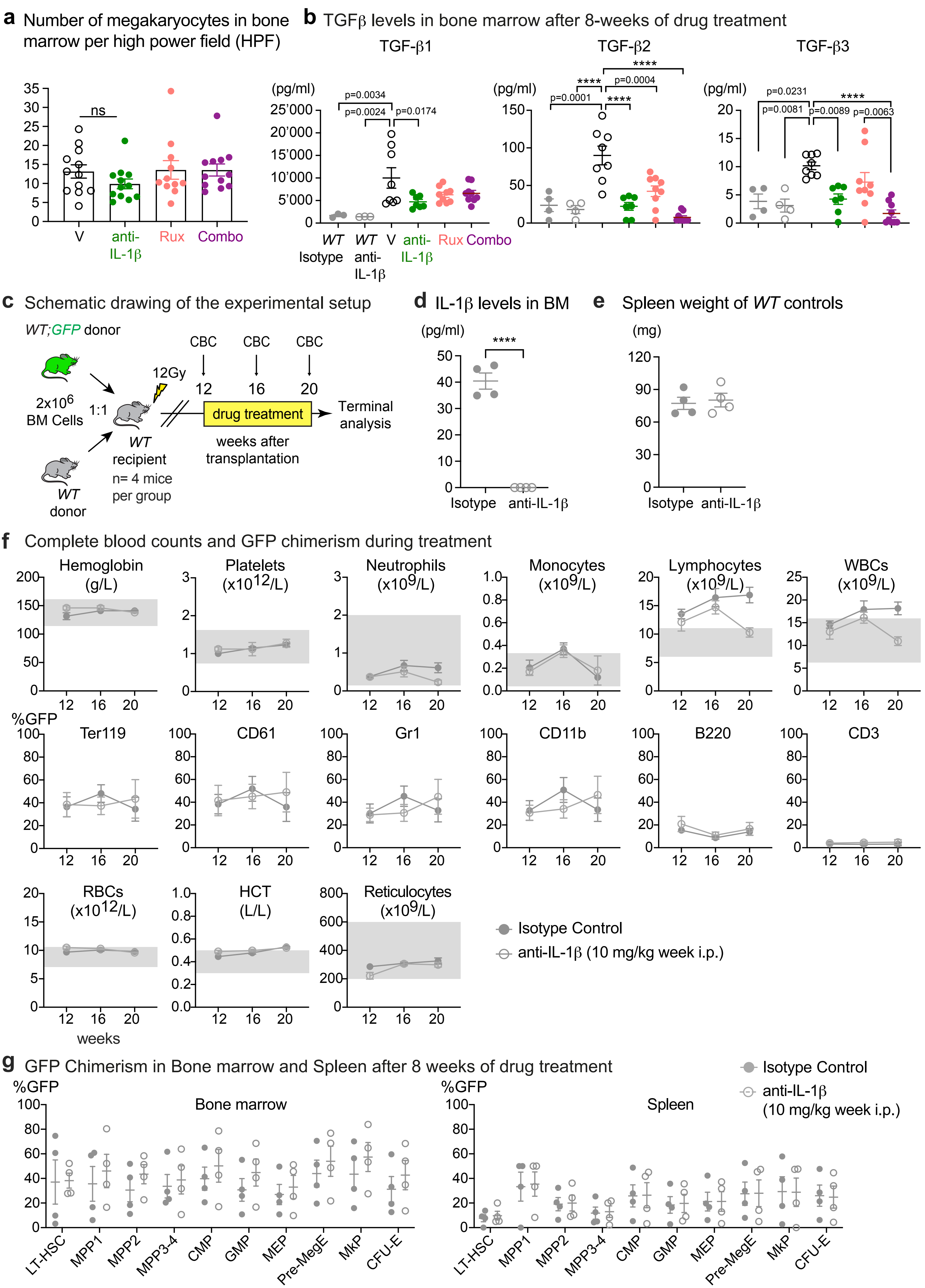

Legend to Supplementary Figure 10

Supplementary Figure 10. Effect of anti-IL-1β treatment on *WT* mice transplanted with *WT* bone marrow cells. **a,** Bar graphs show the number of megakaryocytes per high power visual field (HPF) at magnification 400x in Vehicle (n=12); Rux (n=11); anti-IL-1β (n=12) and combo (n=12) treated mice. Ten HPF were counted for each mouse and each dot represents the average number of megakaryocytes per 10 HPF. **b,**TGF-β levels in bone marrow of Vehicle (n=8); Rux (n=9); anti-IL-1β (n=7) and combo (n=9) treated mice. One-way ANOVA with Tukey's multiple comparison test was performed for statistical comparisons between groups. **c,** Schematic of the experimental setup for the drug treatment and regimen is shown. **d,** IL-1β protein levels in the bone marrow lavage of *WT* mice after 8 weeks of treatment with either isotype or anti-IL-1β antibody (n=4 per group). Two-tailed unpaired t-test was performed for the statistical comparison between groups. **e,** Spleen weights of mice after 8 weeks of drug treatment (n=4 per group). **f,** Complete blood counts and mutant cell (GFP) chimerism in the peripheral blood during drug treatment is shown (n=4 per group) in erythroid (Ter119), megakaryocytic (CD61), granulocytic (Gr1) and monocytic (CD11b) lineages. Red cell parameters are also shown (bottom). Grey area represents normal range. **g,** GFP chimerism after 8-weeks of drug treatment in hematopoietic stem cells (HSCs) and progenitors (HSPCs) are shown in bone marrow and spleen (n=4 per group). All data are presented as mean ± SEM. *P < .05; **P < .01; ***P < .001; ****P < .0001. Source data are provided as a Source Data file.

Supplementary Figure 11 (related to Figure 5)

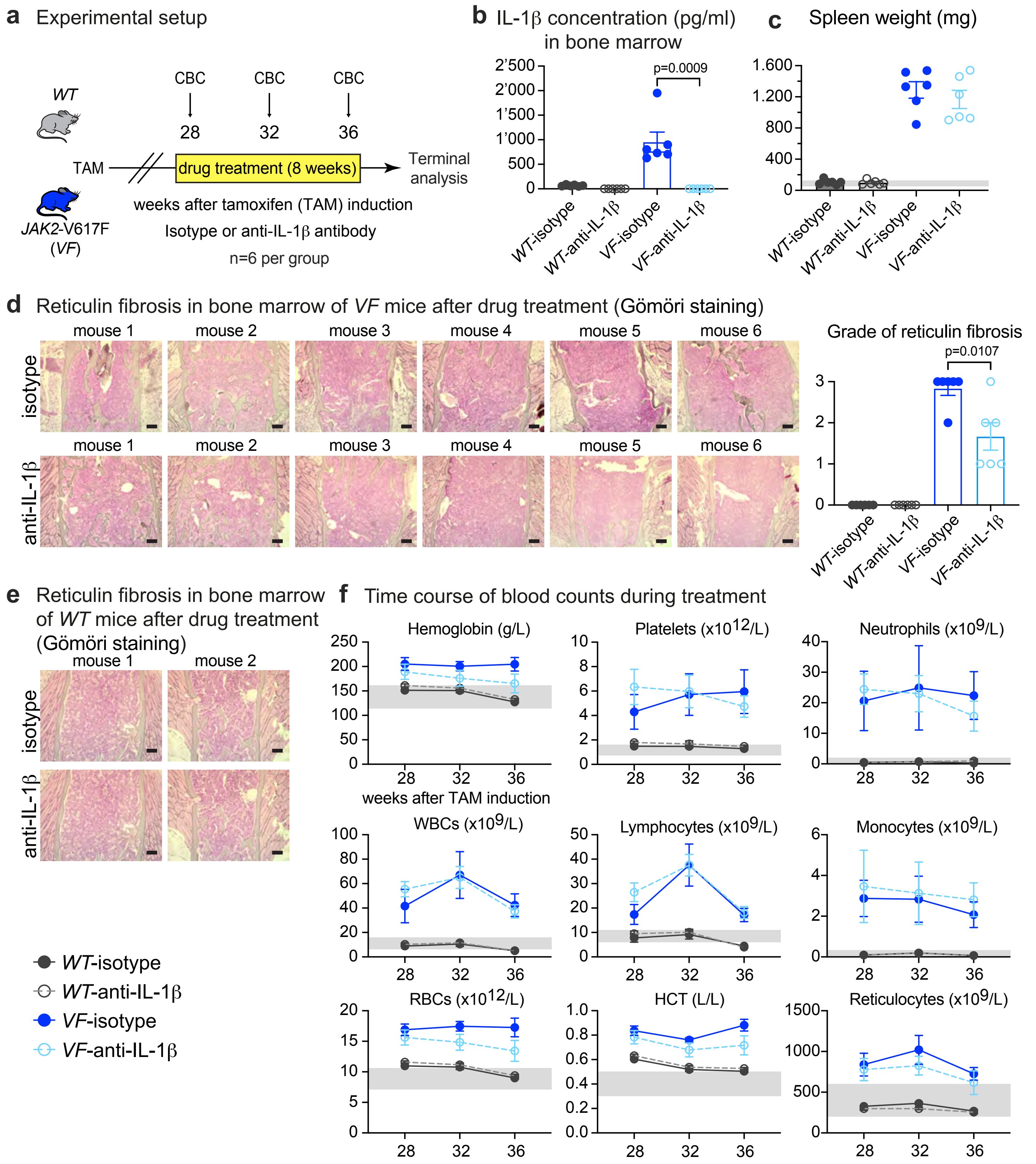

Supplementary Figure 11. Effect of anti-IL-1β antibody treatment on bone marrow fribrosis in non-transplanted MPN mice. a, Schematic drawing of the experimental setup for the drug treatment and regimen. *WT* (n=12) and MPN (*VF*) mice (n=12) were induced with tamoxifen. Treatment with anti-IL-1β antibody or isotype control antibody started 28-weeks after tamoxifen induction. n=6 mice per group. **b,** IL-1β protein levels in the bone marrow lavage of mice (n=6 mice per group) after 8 weeks of treatment with either isotype or anti-IL-1β antibody. Two-tailed unpaired t-test was performed for the statistical comparison between groups.**c,** Spleen weights of mice after 8 weeks of drug treatment (n=6 mice per group). **d,** Representative images of reticulin fibrosis in the bone marrow of each *VF* mice treated with isotype control antibody or anti-IL-1β antibody is shown. Histological grade of reticulin fibrosis is shown (right). Two-tailed unpaired t-test was performed for the statistical comparison between groups. Scale bar is 50 µm. **e,** Representative images of reticulin fibrosis in the bone marrow of *WT* mice treated with isotype control antibody or anti-IL-1β antibody is shown. Scale bar is 50 µm. Similar results were obtained with other mice of each genotype in **d** and **e**. **f,** Complete blood counts of mice (n=6 mice per group) during drug treatment is shown.Grey area represents normal range. All data are presented as mean ± SEM. *P < .05; **P < .01; ***P < .001; ****P < .0001. Source data are provided as a Source Data file.

Supplementary Figure 12 (related to Figure 6)

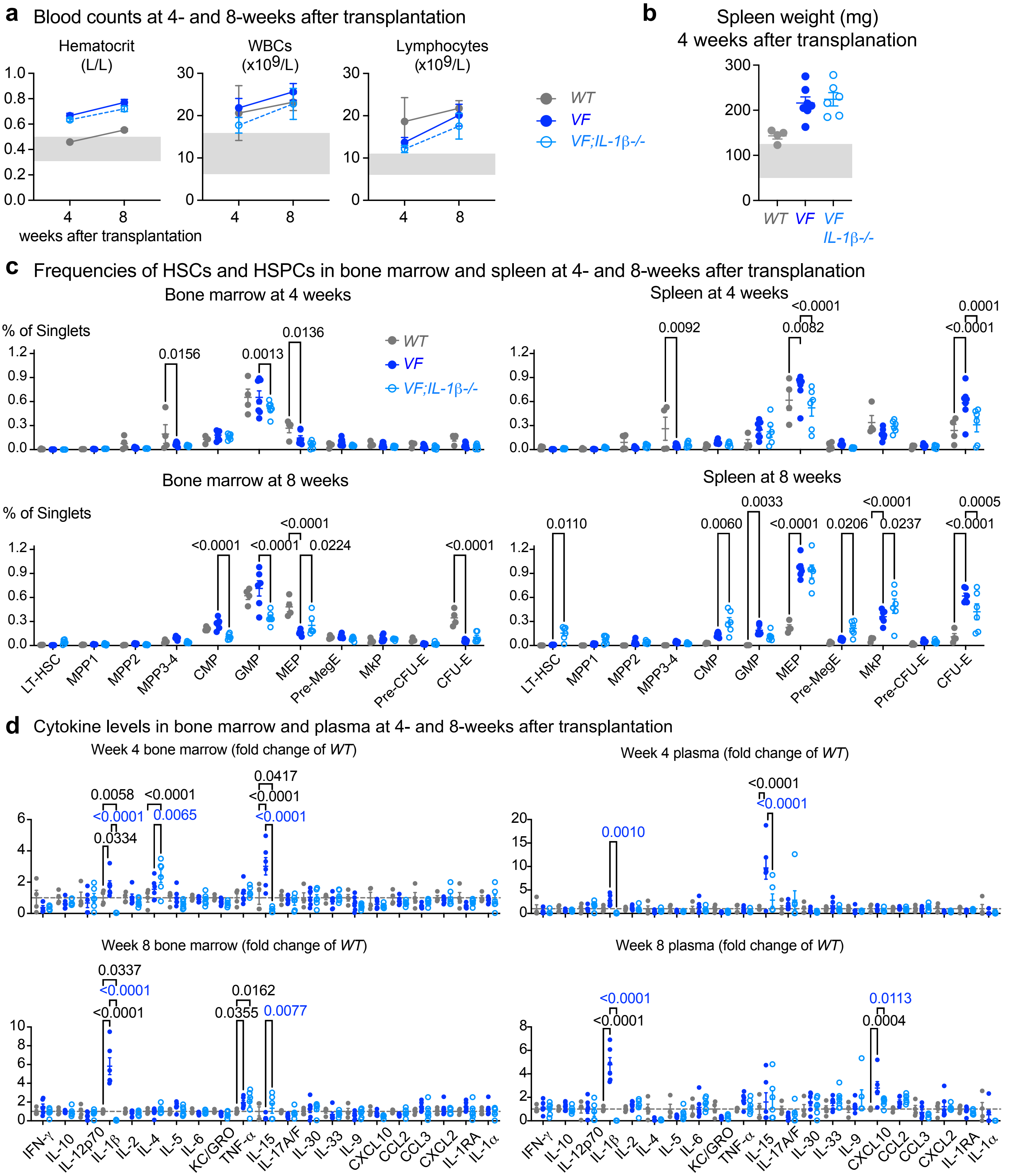

Supplementary Figure 12. Transplantation of *VF* and *VF;IL-1*β$^{-/-}$ BM into *Nestin*-GFP recipients. a, Blood counts at 4-weeks (VF; n=7, VF;IL-1β-/-; n=6, and WT; n=4) and 8-weeks (VF; n=7, VF;IL-1β-/-; n=6, and WT; n=4) after transplantation. **b,** Spleen weight of mice at 4-weeks (VF; n=7, VF;IL-1β-/-; n=6, and WT; n=4) after transplantation is shown. **c,** Frequencies of hematopoietic stem cells and progenitors in bone marrow and spleen at 4-weeks (VF; n=7, VF;IL-1β-/-; n=6, and WT; n=4) and 8-weeks (VF; n=6, VF;IL-1β-/-; n=6, and WT; n=4) after transplantation. Two-Way ANOVA follwed by Dunnetts Multiple comparison test was performed for statistical comparison of groups. Grey shaded area represents normal range. **d,** Multiplex cytokine levels in BM and plasma of mice at 4-weeks (VF; n=6, VF;IL-1β-/-; n=6, and WT; n=4) and 8-weeks (VF; n=6, VF;IL-1 β-/-; n=6, and WT; n=4) after transplantation. Cytokine levels are normalized to WT (dashed line at y=1). Two-way Anova with Tukey's multiple comparison test was performed for statistical analysis. All data are presented as mean ± SEM. *P < .05; **P < .01; ***P < .001; ****P < .0001. Source data are provided as a Source Data file.
