# Supplementary material for: Inhibition of interleukin-1β reduces myelofibrosis and osteosclerosis in mice with JAK2-V617F driven myeloproliferative neoplasm
Source: Nat Commun. 2022 Sep 13;13:5346. doi: 10.1038/s41467-022-32927-4 (PMC9470591; doi:10.1038/s41467-022-32927-4)
Supplement: Supplementary file 3 — Description of Additional Supplementary Files [file 41467_2022_32927_MOESM3_ESM.pdf]

Supplementary Data 1: Information on diagnosis, progression, and gene mutations of MPN patients described in Figures 1 and Supplementary Figures 1-3
