# Supplementary material for: Inhibition of interleukin-1β reduces myelofibrosis and osteosclerosis in mice with JAK2-V617F driven myeloproliferative neoplasm
Source: Nat Commun. 2022 Sep 13;13:5346. doi: 10.1038/s41467-022-32927-4 (PMC9470591; doi:10.1038/s41467-022-32927-4)
Supplement: Supplementary file 5 — Reporting Summary [file 41467_2022_32927_MOESM5_ESM.pdf]

nature portfolio

Corresponding author(s): Radek C. Skoda

Last updated by author(s): Aug 15, 2022

Reporting Summary

Nature Portfolio wishes to improve the reproducibility of the work that we publish. This form provides structure for consistency and transparency in reporting. For further information on Nature Portfolio policies, see our Editorial Policies and the Editorial Policy Checklist.

Statistics

For all statistical analyses, confirm that the following items are present in the figure legend, table legend, main text, or Methods section.

n/a	Confirmed	
☐	☒	The exact sample size (*n*) for each experimental group/condition, given as a discrete number and unit of measurement
☐	☒	A statement on whether measurements were taken from distinct samples or whether the same sample was measured repeatedly
☐	☒	The statistical test(s) used AND whether they are one- or two-sided<br>*Only common tests should be described solely by name; describe more complex techniques in the Methods section.*
☐	☒	A description of all covariates tested
☐	☒	A description of any assumptions or corrections, such as tests of normality and adjustment for multiple comparisons
☐	☒	A full description of the statistical parameters including central tendency (e.g. means) or other basic estimates (e.g. regression coefficient) AND variation (e.g. standard deviation) or associated estimates of uncertainty (e.g. confidence intervals)
☐	☒	For null hypothesis testing, the test statistic (e.g. *F*, *t*, *r*) with confidence intervals, effect sizes, degrees of freedom and *P* value noted<br>*Give P values as exact values whenever suitable.*
☒	☐	For Bayesian analysis, information on the choice of priors and Markov chain Monte Carlo settings
☒	☐	For hierarchical and complex designs, identification of the appropriate level for tests and full reporting of outcomes
☐	☒	Estimates of effect sizes (e.g. Cohen's *d*, Pearson's *r*), indicating how they were calculated

Our web collection on statistics for biologists contains articles on many of the points above.

Software and code

Policy information about availability of computer code

Data collection	BD FACS Diva, Siemens Advia120 Hematology Analyzer using Multispecies Version 5.9.0-MS software (Bayer), Mesoscale Meso Sector S 600 instrument and Discovery Workbench 4.0 software, Nikon Ti inverted microscope and NIS Software, laser scanning confocal microscope (Zeiss LSM 700)
Data analysis	Blood count and organ weights of mice were recorded as indicated in figure legends. Histological staining from sternum/femur, spleen and liver was analyzed by our expert hemato-pathologist who is a co-author(Stefan Dirnhofer). He was completely blinded for the experiemental details. The number of animals and replicates can be found in the respective figure legends. The unpaired two-tailed Student's t-test analysis was used to compare the mean of two groups. Normality tests were performed to test whether the data follows a normal distribution. When the distribution was not normal, non-parametric Mann-Whitney t-tests were performed. For samples with significantly large variances, Welch's correction was applied for t-test. Two-tailed unpaired multiple t-tests with or without correction were also performed for the comparison of multiple groups or one-way or two-way ANOVA analyses followed by Dunn's, Tukey's or Bonferroni's multiple comparison tests were used for multiple group comparisons. Survival rate in mouse experiments was represented with Kaplan-Meier curves and significance was estimated with the log-rank test. Data were analyzed and plotted using Prism software version 7.0 (GraphPad Inc.). FACS data were analyzed using FlowJo (version 10.7.1) software. All data are represented as mean ± SEM. Significance is denoted with asterisks (*p<0.05, **p<0.01, ***p < 0.001, ****p < 0.0001).

For manuscripts utilizing custom algorithms or software that are central to the research but not yet described in published literature, software must be made available to editors and reviewers. We strongly encourage code deposition in a community repository (e.g. GitHub). See the Nature Portfolio guidelines for submitting code & software for further information.

Data

Policy information about availability of data

All manuscripts must include a data availability statement. This statement should provide the following information, where applicable:

- Accession codes, unique identifiers, or web links for publicly available datasets
- A description of any restrictions on data availability
- For clinical datasets or third party data, please ensure that the statement adheres to our policy

All data generated or analyzed during this study are included in this manuscript (and its supplementary information files). Data availability statement is included in the manuscript and source data file is also provided.

Field-specific reporting

Please select the one below that is the best fit for your research. If you are not sure, read the appropriate sections before making your selection.

- [x] Life sciences
- [ ] Behavioural & social sciences
- [ ] Ecological, evolutionary & environmental sciences

For a reference copy of the document with all sections, see nature.com/documents/nr-reporting-summary-flat.pdf

Life sciences study design

All studies must disclose on these points even when the disclosure is negative.

Sample size	Sample size was determined based on published research in the field and our own published research (Rao et al, Blood 2019 and Rao et al, Blood 2021). The sample sizes used were sufficient to demonstrate large differences between experimental conditions.
Data exclusions	For the in vivo studies, mice were excluded from analysis if they were sacrificed or died for reasons unrelated to MPN-phenotype or treatment
Replication	Experiments involving statistical analyses were performed using at least three independent biological replicates, or using samples from at least three different patients/individuals or from at least three different mice for each experimental condition. Sample size for animal studies were large enough to justify replication and reproducibility. In other experiments, all attempts at replication were successful
Randomization	Mice in the drug treatment cohort were randomized based on peripheral blood counts (mainly platelet counts) and mutant cell chimerism in the peripheral blood (GFP expression). All other samples and animals were analysed and allocated randomly.
Blinding	The hemato-pathologist was completely blinded for experimental or sample details to analyze or grade histology sections from mice. All other experiments were performed in a non-blinded manner, because the experimental design was complicated and blinding feasibility was poor.

Reporting for specific materials, systems and methods

We require information from authors about some types of materials, experimental systems and methods used in many studies. Here, indicate whether each material, system or method listed is relevant to your study. If you are not sure if a list item applies to your research, read the appropriate section before selecting a response.

Materials & experimental systems

n/a	Involved in the study	
[ ]	[x]	Antibodies
[x]	[ ]	Eukaryotic cell lines
[x]	[ ]	Palaeontology and archaeology
[ ]	[x]	Animals and other organisms
[ ]	[x]	Human research participants
[x]	[ ]	Clinical data
[x]	[ ]	Dual use research of concern

Methods

n/a	Involved in the study	
[x]	[ ]	ChIP-seq
[ ]	[x]	Flow cytometry
[x]	[ ]	MRI-based neuroimaging

Antibodies

Antibodies used	Fc receptors (1:20; 564220, BD)<br>lineage-FITC (1:20; 348701) BioLegend<br>CD34-Pacific Blue (1:100; 343512) BioLegend<br>CD38-APC (1:50; 356606) BioLegend<br>CD123-BV605 (1:100; 306026) BioLegend<br>CD41-PE-Cy5 (1:50; 343512) BioLegend

CD45RA-BV786, (1:50; 563870; BD biosciences)
IL-1R1-PE (1:20; FAB269P), IL-1RAcP-PE (1:20; FAB676P) or isotype goat IgG-PE antibody (1:20; IC108P) from R&D systems
CD4 (1:200; 100404, clone GK1.5), BioLegend
CD8 (1:200; 100704), BioLegend
B220 (1:200; 103204), BioLegend
TER-119 (1:100; 116204), BioLegend
CD11b (1:400; 101204), BioLegend
Gr-1(1:400; 108404) from BioLegend
Sca-1-APC-Cy7 (1:100; 108126), BioLegend
CD117 (c-kit)-BV711 (1:100; 105835), BioLegend
CD48-AF700 (1:100; 103426), BioLegend
CD150 (SLAM)-PE-Cy7 (1:100; 115914), BioLegend
CD16-PE (1:100; 101308), BioLegend
CD41-BV605 (1:100; 133921), BioLegend
CD105-PerCP-Cy5.5 (1:100; 120416) from BioLegend
CD34-AF647 (1:25; 560230; BD biosciences)

Sytox Blue; 1:1000; S34857 Thermo Fisher Scientific;

Streptavidin-Pacific Blue; 1:100; S11222 Thermo Fisher Scientific

Mouse IgG2a isotype control antibody and mouse IgG2a anti-mouse IL-1β antibody (01BSUR) (Osborn et al, Cytokine 2008; Gomez et al, Nature Medicine 2018; Potus et al, Circulation 2020) (Both provided by Novartis Pharma AG (Basel, Switzerland).

This information is also included in Methods section of the manuscript.
Antibody catalogue number are exclusive and only includes single clones (can easily be found on the website of manufacturer).

Validation

All antibodies were used according to their manufacturer's instructions. This information can be found directly on the website of these manufacturers with the catalogue numbers that are already described in this reporting summary and also Methods section of the manuscript:
https://www.biolegend.com/fr-ch
https://www.rndsystems.com
https://www.bdbiosciences.com/en-ch
https://www.abcam.com
https://www.thermofisher.com/ch/en/home.html

Animals and other organisms

Policy information about studies involving animals; ARRIVE guidelines recommended for reporting animal research

Laboratory animals

All mice were of pure C57BL/6N background, and kept under specific pathogen-free conditions with free access to food and water in accordance to Swiss federal regulations and mice were maintained in 12 hour light/dark cycle with 23 degree celsius ambient temperature and 40% humidity. All mice stains used in this study are described in Methods section of the manuscript.

Wild animals

No wild animals were used in the study

Field-collected samples

No field collected samples were used in the study

Ethics oversight

Ethical approvals were obtained for all experiments with laboratory animals from Cantonal Veterinary Office of Basel-Stadt, Switzerland. This information is also included in the manuscript.

Note that full information on the approval of the study protocol must also be provided in the manuscript.

Human research participants

Policy information about studies involving human research participants

Population characteristics

Blood samples and clinical data of MPN patients were collected at the University Hospital Basel, Switzerland. Written informed consent was obtained from all patients in accordance with the Declaration of Helsinki. The diagnosis of MPN was established according to the 2016 revision of the World Health Organization classification of myeloid neoplasms and acute leukemia. Molecular diagnosis of patients are specified in Supplementary Data 1.

Recruitment

Only frozen materials such as PBMCs and serum from patients that were recruited for our observational cohort study were used.

Ethics oversight

The study was approved by Ethik Kommission Beider Basel, Switzerland

Note that full information on the approval of the study protocol must also be provided in the manuscript.

Flow Cytometry

Plots

Confirm that:

- [x] The axis labels state the marker and fluorochrome used (e.g. CD4-FITC).
- [x] The axis scales are clearly visible. Include numbers along axes only for bottom left plot of group (a 'group' is an analysis of identical markers).
- [x] All plots are contour plots with outliers or pseudocolor plots.
- [x] A numerical value for number of cells or percentage (with statistics) is provided.

Methodology

Sample preparation	Frozen PBMCs from MPN patients and normal controls were thawed and stained after blocking Fcg receptors (#564220, BD) with following human antibodies: lineage-FITC, CD34-Pacific Blue, CD38-APC, CD123-BV605 and CD41-PE-Cy5 (BioLegend), CD45RA-BV786, (BD biosciences) and IL-1R1-PE or IL-1RAcP-PE (R&D systems). Mouse BM cells were harvested from long bones (2 tibias and 2 femurs) by crushing bones with mortar and pestle using staining media (Dulbecco's PBS+ 3% FCS+ pen/strep). Cells were filtered through 70µm nylon mesh to obtain a single-cell suspension. Total spleen cells were harvested by crushing the spleen against 100 µm cell strainer. Red blood cells were lysed (ACK buffer, Invitrogen) and stained with following antibodies for FACS analysis: A mixture of biotinylated monoclonal antibodies CD4, CD8, B220, TER-119, CD11b, and Gr-1 was used as the lineage mix (Lin). Sca-1-APCCy7, CD117 (c-kit)-BV711, CD48-AF700, CD150 (SLAM)-PE-Cy7, CD34-AF647, CD16-PE, CD41-BV605, CD105-PerCP-Cy5.5 (all from BioLegend).
Instrument	Cells were analyzed on a Fortessa Flow Cytometer (BD biosciences)
Software	Data acquired using FACS Diva (BD Biosciences) and analyzed using FlowJo (version 10.7.1) software.
Cell population abundance	Around 1000-5000 HSCs, pre-MegE and MkP were directly sorted in RNA lysis buffer (PicoPure, Thermo Scientific) for RNA extraction. The quality of purified RNA was verified by Agilent Bioanalyzer (details are provided in Methods).
Gating strategy	Using the FSC/SSC gating, debris was removed by gating on the main cell population. Sytox-Blue or Green (Invitrogen) was used to exclude dead cells during FACS analysis. Live, singlet cells were selected for gating. Doublets were excluded using FSC-width (W) and height (H) as well as SSC-W and H. Hematopoietic progenitors and stem cells were gated according to published literature and positive gates were defined based on isotype/unstained/FMO controls. Gating strategies and cutoffs are described in Supplementary figures.

- [x] Tick this box to confirm that a figure exemplifying the gating strategy is provided in the Supplementary Information.
